# Surgical Management of the Drooling Child

**DOI:** 10.1007/s40136-018-0188-2

**Published:** 2018-03-20

**Authors:** Rachael Lawrence, Neil Bateman

**Affiliations:** 1 Specialist Registrar in ENT, Queen’s Medical Centre, Nottingham, NG7 2UH UK; 2 Paediatric ENT, Royal Manchester Children’s Hospital, Oxford Road, Manchester, M13 9WL UK

**Keywords:** Drooling, Sialorrhoea, Paediatric, Management, Surgical

## Abstract

**Purpose of Review:**

Our goal is to present the most up-to-date options in the surgical management of drooling in the paediatric population. While the clinical assessment of the drooling child and conservative management options are discussed, this review focuses on the most recent evidence for surgical interventions to treat drooling in children.

**Recent Findings:**

In terms of advances in the management of drooling, further experience and outcomes with the use of botulinum toxin injections is discussed. Moreover, the latest evidence-base for salivary duct ligation and relocation procedures are presented. Finally, the trans-oral approach to submandibular gland excision for the management of drooling may gain popularity through the aim of reducing surgical morbidity.

**Summary:**

The drooling child should be managed with an evidence-based stepwise approach delivered by a multidisciplinary team (MDT). Children with normal neurological development should be treated conservatively through parental reassurance. There are numerous interventions available for the drooling child with impaired neuromuscular development. When conservative measures fail, treatment options include botulinum toxin injections and surgical procedures such as salivary duct ligation, salivary duct relocation and salivary gland excision. Management must be targeted to the individual needs and comorbidities of the child to maximise treatment outcomes.

## Introduction

Sialorrhoea is defined as the spillage of saliva due to either the excessive production of saliva (primary sialorrhea) or decreased frequency of swallowing (secondary sialorrhea) [1, 2]. It is more commonly referred to as drooling and can be further classified as either anterior or posterior. Anterior drooling is when saliva leaves the oral cavity. Posterior drooling results in secretions pooling in the hypopharynx thereby increasing the risk of aspiration pneumonia. As the orofacial skeleton and swallowing mechanism mature, drooling tends to resolve in children without neuromuscular comorbidities. Neuromuscular disorders impact upon this maturation process and may cause drooling to persist beyond the age of 5 years, after which it is considered abnormal. In addition to social implications, drooling may also cause skin irritation, a requirement for numerous clothing changes and in more severe cases dehydration and aspiration pneumonia.

The management of drooling requires a thorough clinical assessment with a multidisciplinary team (MDT) approach to treatment, options for which include behavioural, pharmacological and surgical interventions. Behavioural approaches are based on physiotherapy and speech therapy, but are reported to be associated with significant relapse rates because of the high levels of motivation and time required [3]. Pharmacotherapy with anticholinergic medications may cause side effects such as dry mouth, blurred vision and urinary retention. Hence, other management options such as the injection of botulinium toxin and surgical approaches such as salivary duct ligation, relocation and/or gland excision are often utilised. In this article, we give an overview of all the established treatment options for drooling; however, our discussion concentrates on the evidence-based efficacy and safety of botulinum toxin injection and all available surgical interventions.

## Clinical Assessment

Rating systems for the severity of drooling exist, but are not commonly used in clinical practice. They include the Blasco’s scale [4] and the Teachers’ Drooling Score [5]. A history from a parent is often more useful in the clinical context and this should include enquiries about the number of bib or clothing changes. The presence of skin changes or infection may also reflect the severity of the problem and help to establish the impact on the child, family and carers. A socially aware child may be significantly embarrassed and in severe cases drooling may have negative psychosocial consequences. When managing a less aware child, parental expectations and the benefits and risks of any treatment must be carefully balanced.

The neurological status of any child presenting with drooling is also important to establish. The child’s swallowing abilities are important in assessing likely causes of drooling and also in considering surgical interventions. Children with speech problems and difficulty handling food and saliva in the mouth may suffer from oral motor dyspraxia [6]. Coughing and choking episodes in addition to recurrent chest infections suggest aspiration may be a problem. Gastro-oesophageal reflux can lead to hyperstimulation of the salivary glands and is commonly associated with neuromuscular disorders [6]. A medication history is vital as those with cholinergic effects, such as anticonvulsants, may also lead to hypersalivation.

An examination of the patient should include an assessment of the oral cavity. Dental pathology such as caries, malocclusion and gingivitis can all contribute to drooling. Poor head posture and position in addition to an open-mouth posture due to nasal obstruction also exacerbates drooling.

## Management

For normally developing children under the age of 5 years, simple reassurance for the parents is sufficient. Other children are best managed as part of a MDT which may include a paediatrician, a speech and language therapist, a paediatric dentist and an otolaryngologist [6]. Little et al. 2009 suggest using an evidence-based, stepwise approach to overall management of the drooling child and involves the following stages: (i) review of posture and positioning, (ii) oral awareness and oral motor skills training, (iii) orthodontic treatment, (iv) pharmacotherapy, (v) botulinum toxin and (vi) surgical intervention.

### Conservative Management

Problems with posture and positioning may be addressed and improved with physiotherapy input and advice with regard to items such as wheelchairs which affect positioning. Where there is mouth breathing as a result of nasal obstruction, consideration should be given to addressing this. A dental opinion may be able to address the issue of dental caries and malocclusion.

Oral awareness and oral motor skills training with the involvement of a speech and language Therapist may be appropriate for children who are able to cooperate, and it has been suggested that this be tried for a least 6 months before considering surgical intervention [7]. In clinical practice, this is often impractical. Increased tongue movement and an improved swallow may be achieved by palatal training appliances; however, high-level evidence on their efficiency is lacking [8].

Anticholinergic medication can block the parasympathetic innervation to the salivary glands that stimulates saliva production. While this is often effective, however, side effects such as blurred vision, urinary retention and hyperactivity can limit its clinical usefulness.

#### Botulinum Toxin A

Botulinum toxin is a potent neurotoxin, produced by the *Clostridium botulinum* bacterium that inhibits the release of acetylcholine at the presynaptic neurosecretory junctions within the salivary glands. It does so by permanently binding with the channel protein responsible for acetylcholine transport. The blockade, although irreversible, has only a temporary effect as new nerves grow to create new neural connections [9]. Injection of the salivary glands with botulinum toxin A has been used as an alternative to surgical approaches.

There is a general agreement amongst authors that injection of Botox should be carried out under ultrasound guidance. There is a variation of practice and opinion as to which glands should be injected, the dose of Botox and the volume of injection used. Guidelines published by the Starship Hospital, Aukland, NZ [10••], recommend (using Allergan Botox preparation) titrating the dose on an individual basis but as a general rule:

- Maximum dose per submandibular gland of 20 U
- Maximum dose for both submandibular glands of 40 U
- Maximum dose per parotid gland of 10
- Maximum dose for both parotid glands of 20 U
- Total maximum dose per patient of 60 U.

They suggest that in smaller and/or younger children (i.e. < 10 years or < 20 kg) a smaller dose be considered (for instance 10–15 U per submandibular gland). A dilution of 100 U in 1 ml is recommended. They recommend that, in the first instance, only the submandibular glands are injected. The requirement for injection of the parotid glands should be considered in children with a limited response to submandibular injection.

At the time of writing, the most recently published evidence on botulinum toxin A injection of salivary glands for drooling described a prospective study involving 17 patients aged 12.1 ± 5.1 [4–19] years, all of whom had neurological impairment [11]. Botulinum toxin A (BOTOX ®) was injected into the parotid (30 U) and submandibular glands (20 U) bilaterally by the same radiologist under ultrasound control and general inhalational anaesthesia (GA). The outcome was evaluated through the DSFS Drooling Severity (S) and Frequency (F) Scales that was applied before treatment and at 1-, 3-, and 6-month after injection. The scale runs from 1 (best) to 5 (worst) for severity (S) and 1 to 4 for frequency (F). Treatment was considered a failure when the score did not improve at the 1-month evaluation. A deterioration of 3- or 6-month scores to the pre-treatment score was considered a recurrence. The pre-treatment S + F score was 8.59 ± 0.71; which decreased significantly to 4.65 ± 2.32 (*p* = 0.001) at the 1-month post-injection evaluation. The 3- and 6-month scores were also significantly lower than the pre-treatment score (4.00 ± 1.96, *p* = 0.002; 5.36 ± 2.20, *p* = 0.005; respectively), but there was an increase between the 3- and 6-month evaluations (*p* = 0.01). With a follow-up of 20.1 ± 9.2 [4–36] months, 4 out of the 13 successful injections needed a second injection after 7.5 ± 3.1 [3–10] months. The patient with the longest time not requiring re-injection had 28 months of follow-up. All but two (88%) of parents/caregivers said they would repeat the treatment. In terms of complications, one patient (6%) experienced mild dysphagia that spontaneously regressed.

Alvarenga et al., 2017 reported the efficacy of botulinum toxin A injection for drooling to be in line with other previously published paediatric and adult studies (Table 1). Although the procedure is usually performed under GA, evidence shows it to be an effective, minimally invasive treatment for drooling that is associated with few complications. The most favourable results have been shown to occur around 3 months after injection; with a requirement for re-injection becoming apparent at around 6 months [11, 12]. Although some studies have utilised the same injection protocol [11, 12], the ideal dosage and frequency of injections may need clarification through further studies. However, this may be obviously vary amongst paediatric patients of different age and weight.

**Table 1:** Studies undertaken to investigate the efficacy and safety of botulinum A injection for drooling

Author(s) and reference	Number and age range of patients	Neurological status of patients	Botulinum toxin protocol	Outcome measure and results	Follow-up period	Complication rate
Meece et al. 2010 [12]	6 (2–17 years)	Neurological impairment	Bilateral parotid (30 UI) and SMG (20 UI) of botulinum toxin A with ultrasound guidance under GA	Telephone interview of parent: drooling reduced in 2–12 days after injection in all 6 patients; effect lasting 3–5 months in 5 patients	Unclear	1 patient experienced transient swelling around injection site, nil else
Breheret et al. 2010 [13]	70 (1–84 years)	Neurological impairment	Various protocols were applied as a function of the patient. The dose in children was 5 mU/kg (max 100 mU) of botulinum toxin A with US guidance under GA. Both SMG and parotid glands were injected	Telephone interview of parent/relative. Overall beneficial effect reported in 6% of cases; no efficacy in 25.4%, partial efficacy in 22.5%, brief efficacy (< 1 month) in 1.8% and good efficacy (resolution of symptoms) in 42.3%	6–8 weeks	No major complications reported
Matthew et al. 2016 [14]	111 (4 months to 34 years)	Neurological impairment	Bilateral parotid and SMG gland botulinum A injections with US guidance under GA. Weight based protocol 15 U/gland for < 15 kg, 20 U/gland for 15-25 kg and 25 U/gland for > 25 kg	Telephone interview of caregiver. Overall treatment effectiveness was 68%; 41% experienced very effective results, 31% experienced resolution for > 2 months, 25% no benefit	Average 3 years [2 days–9 years]	No major complications, minor rate < 2%
Jongerius et al. 2003 [15]	44 (age not reported)	Neurological impairment	Bilateral SMG injection with US guidance under GA. A total dose of 30 to 50 U was used	Evaluation with repeated salivary flow measurement from the salivary glands at regular intervals up to 24 weeks after injection. Exact effect not described ‘the majority of patients showed a positive effect of injections’	Unclear	No major complications. A ‘few’ patients experienced temporary swelling of the SMG gland

### Surgical Management

Surgical interventions for drooling may be considered when conservative measures or botulinum toxin injections fail to improve or control the symptoms and consequences of drooling. Surgical treatments may be utilised either alone, in combination or succession.

#### Salivary Duct Ligation

The concurrent ligation of the parotid and submandibular ducts (4-duct ligation) has been described in the literature as a simple and minimally invasive first-line surgical option for the management of drooling. The attractiveness of the procedure lies in its surgical simplicity, lack of external skin scars and avoidance of nerve injury. Studies undertaken to examine the treatment of drooling with the 4-duct ligation procedure have been both prospective [16] and retrospective [1, 17–19, 21, 22] in design. Success rates vary between 30 and 100%. Recurrence rates are reported to range from 0 to 69% with the timing of recurrence after the procedure ranging from 3.5 to 9 months.

The most recently published study utilising 4-duct ligation is a retrospective cohort study that was performed in a tertiary paediatric centre. This study included 38 children with neurological impairment [1]. The median age was 11 years (age range 5–17 years). The mean (SD) duration of effect was 52.6 (20.4) months. Thirteen complications were documented in 12 patients. The most common complications were persistent facial/glandular swelling and aspiration pneumonia, the causative reasons for which are not elaborated upon by the authors. Eighty percent (28 of 35) of caregivers reported an improvement in their child’s drooling at 1 month, while 69% (25 of 36) and 71% (24 of 34) stated that there was an improvement at the 1 year and the most recent follow-up. Other established paediatric studies have also analysed the treatment of drooling with the 4-duct ligation procedure. Some of these studies analysed improvements in symptoms of anterior drooling only [19], whereas others examined both anterior and posterior drooling symptom control [20]. El-Hakim et al. 2008 noted a significant improvement in anterior and posterior drooling with a reduction in aspiration pneumonia. In this study, some patients underwent 2-duct ligation (bilateral submandibular duct ligation) and others 4-duct ligation (bilateral submandibular and parotid duct ligation). When the 2- vs 4- duct ligation procedures were examined in relation to drooling control and Glasgow Children’s Benefit Inventory (GCBI) scores, a weak correlation was found. However, the authors concluded that due to the small number of patients in each arm of the study, a strong case for 4-duct over 2-duct ligation could not be made. Yet, they did recommend a 4-duct ligation procedure where the primary complaint is aspiration. There were no reported complications in this patient series.

A prospective study by Scheffer et al. 2013 divided neurologically impaired children into three subgroups: (i) anterior drooling, (ii) anterior drooling and limited posterior drooling and (iii) posterior drooling [20]. Children in the study underwent either ligation of both submandibular ducts (2-duct ligation), both submandibular ducts and a single parotid duct (3-duct ligation), or 4-duct ligation. Four-duct ligation was reserved for children with severe posterior drooling. Fifteen children underwent 2-duct ligation, three children underwent 3-duct ligation and four children underwent 4-duct ligation. Results showed a highly significant reduction in anterior drooling, with both the drooling quotient and caregiver visual analogue score reducing after both 2 and 8 months follow-up. Although not a primary outcome measure, 3-duct ligation did not show a greater improvement over 2-duct ligation for anterior drooling. Although the number of aspiration pneumonias due to posterior drooling appeared to decline, this was not statistically significant for any of the duct ligation procedures and was perhaps due to the relatively low number of children with aspiration pneumonia included in this particular study.

The technique of salivary duct ligation may offer a simple, effective and minimally invasive approach to the management of drooling in children. Unfortunately, other additional surgical procedures are still frequently required after salivary duct ligation techniques due to a significant recurrence rate of drooling [21]. Future higher-quality studies such as randomised control trials are required to further assess the effectiveness of this technique. Appropriate patient selection for 2- through to 4-duct ligation also needs further consideration and evidence-base. This selection process must take into account whether a patient demonstrates anterior or posterior drooling, or indeed a combination of both. Salivary duct ligation appears to be a safe technique overall, although there are reports of complications including xerostomia with an increased risk of dental caries, facial swelling, delayed reinitiation of oral feeding and aspiration pneumonia [1]. Ranula formation, a common complication of submandibular duct transposition, has been reported by some authors as unlikely in the procedure of salivary duct ligation through lack of interruption of the sublingual ducts [17].

#### Salivary Duct Relocation

As the submandibular glands produce approximately 70% of the resting salivary output, they are the obvious target for surgical intervention in terms of diversion of saliva. Submandibular duct relocation (SMDR) was first described by Laage-Hellman in 1969 [23] and has been shown to be effective in about 80% of children who have a safe swallow [24–29]. The surgery involves the bilateral relocation of the submandibular ducts to the inferior pole of the palatine tonsil.

The most recently published study that has analysed the impact of SMDR on drooling is that by Kok et al. 2016. This prospective cohort study included 72 children and adolescents with neurodevelopmental disabilities affected by moderate to severe drooling. Mean age at the time of surgery was 15 years 2 months (SD 4 years 3 months). A caregiver questionnaire to document the impact of drooling was administered before, and 8 and 32 weeks after surgery. All children were reviewed by a speech and language therapist and appeared to have a safe pharyngeal swallow function. Following bilateral SMDR and sublingual gland excision, the mean Visual Analogue Score (VAS, 0–100) scores demonstrated a significant (*p* < 0.001) reduction in the severity of drooling from 81 at baseline to 28 and 36 after 8 and 32 weeks, respectively. This was accompanied by a decrease in the amount of daily care required and reduced economic consequences in addition to increased social contact with other children and adults after surgery. In terms of complications, four children required prolonged intubation due to transient swelling of the floor of the mouth, three children developed pneumonia and one child required tube feeding for 3 days. However, all complications were said to resolve without residual problems.

In the aforementioned study, the sublingual glands were also removed which aims to prevent ranula formation, a complication reported to occur in 8–9% of cases [24, 25]. However, dissection and removal of the sublingual glands requires more extensive floor of mouth dissection, prolonged surgical and anaesthetic time and hence potentially increased morbidity. Glynn and O’Dwyer, 2007 prospectively assessed whether SMDR alone produced the same success rates for drooling. They found no statistical difference in drooling scores between SMDR alone and SMDR and sublingual gland excision, yet the post-operative haemorrhage rate and pain scores were higher when the sublingual glands were also excised.

Numerous studies have shown SMDR with or without sublingual gland excision to be highly effective. However, this technique is only indicated for anterior drooling as it will obviously exacerbate any posterior drooling associated with aspiration. It is therefore vital to enquire about any history of choking with liquids, any required dietary modifications and any recurrent lower respiratory tract infections. If there is any concern about aspiration, then a formal swallowing assessment by a specialist speech and language therapist should be organised. This will ensure the presence of a safe swallow, which is essential for patients that are being considered for SMDR. As previously discussed, the procedure may be associated with morbidity; hence, patients and carers should be appropriately counselled. Bilateral parotid duct diversion has also been reported in the literature. Although reported to improve subjective outcomes for anterior drooling, it has usually been performed in conjunction with another surgical intervention for drooling [2]. When performed alone, patient numbers have been small so it is difficult to draw definitive conclusions on effectiveness [30–33].

#### Salivary Gland Excision

As already discussed, SMDR is contraindicated in posterior drooling, and the effectiveness of 4-duct ligation in reducing aspiration is not entirely clear. Bilateral submandibular duct excision is another potential surgical intervention suitable for the management of both anterior and posterior drooling.

Delsing et al. 2015 analysed 45 neurologically impaired children and adolescents who had undergone bilateral submandibular gland excision (SMGE) as their only surgical procedure [34]. The average age at the time of surgery was 15.6 years (SD 6.72, range 2–38 years). A pre-operative assessment by a speech and language therapist revealed that 39% had anterior drooling only whilst 61% had both anterior and posterior drooling. In terms of success, the authors reported an overall response rate of 63% with significantly improved subjective outcome measures following surgery. Drooling intensity was evaluated using the drooling quotient (DQ), which is a validated, semi-quantitative direct observational method. The DQ is expressed as a percentage estimated from the ratio of observed drooling episodes and the total number of observations (DQ [%] = 100 × number of drooling episodes/20). Successful therapy effect was defined as a higher than 50% reduction compared to baseline. The DQ was reduced from a baseline score of 33.5 to 17.1 after 8 weeks and 9.9 after 32 weeks (*p* = 0.002). In terms of complications, two patients required an admission to the intensive care unit following a post-operative haemorrhage requiring repeat surgical intervention. One patient reported xerostomia. No procedures resulted in a marginal mandibular, lingual or hypoglossal nerve palsy.

Parotid duct ligation in addition to SMGE has also been reported in the literature. Removal of the submandibular gland is said to eliminate resting salivary flow in the majority of children, while ligation of the parotid ducts eliminates the major source of mastication induced salivary production [35]. A case series involving 93 children who underwent bilateral SMGE with parotid duct ligation showed 87% of caregivers reported either no further drooling or a significant improvement in drooling [35]. However, parotid duct ligation carries the risk of parotid gland swelling and infection. In the aforementioned case, series one patient had bilateral swelling that resolved spontaneously and one had a unilateral sialadenitis that resolved after a course of an intravenous antibiotic. The additional risk of parotid duct ligation in addition to SMGE must be considered and the patient and parent/caregiver appropriately counselled.

SMGE is traditionally performed by the trans-cervical approach. However, a recent report describes the procedure being performed via the trans-oral route avoiding some risks such as a cervical scar and injury to the marginal mandibular branch of the facial nerve. Hughes et al. 2017 retrospectively reviewed ten patients aged 9 to 17 years who underwent trans-oral submandibular gland excision for various indications [36]. No patient was reported to suffer vessel or nerve injury. The authors have no experience of this surgical technique.

Reed et al. 2009 performed a meta-analysis of the surgical management of drooling with a random-effects modelling estimating that the overall subjective success rate for all surgical procedures to be 81.6% (95% CI, 77.5–85.7%, *p* < .001) [2]. Bilateral SMGE and parotid duct rerouting appeared to have the highest subjective success rates at 87.8% (CI 80.5–95.1%, *p* < .001) and 4-duct ligation the lowest at 64.1% (CI 27.6–100%, *p* = .001). However, the authors concluded that studies were generally low quality and heterogeneous. Further high-level quality evidence to assess the indications, patient selection, effectiveness and safety of all the aforementioned surgical techniques is therefore very much called-for.

## Conclusion

The drooling child should be managed with an evidence-based stepwise approach delivered by a multidisciplinary team (MDT). Children with normal neurological development should be treated conservatively through parental reassurance. For the drooling child with impaired neuromuscular development, where conservative measures fail, other treatment options include botulinum toxin injections and surgical procedures such as salivary duct ligation, salivary duct relocation and salivary gland excision. Evidence shows botulinum toxin injection and surgical interventions to be successful in terms of improving subjective outcome measure. Differences in success rates can be explained by the multiple causes of drooling and the heterogeneity of the population. Ligation of the salivary ducts has gained popularity due to the simplicity of the procedure and its low risk of morbidity; however, outcomes are variable and further procedures are often required to control symptoms. Salivary duct relocation procedures have demonstrated effectiveness in anterior drooling but this is dependent on a normal swallow and is contraindicated in children with posterior drooling. Bilateral SMGE is effective and morbidity may potentially be reduced by a trans-oral approach, although the evidence for this is based on a small number of cases from a single centre at present. Further high-level quality evidence to assess the indications, patient selection, effectiveness and safety of all the aforementioned surgical techniques is still required to further improve individual patient outcomes.
